# Autism, Context/Noncontext Information Processing, and Atypical Development

**DOI:** 10.1155/2011/681627

**Published:** 2011-08-14

**Authors:** John R. Skoyles

**Affiliations:** Centre for Mathematics and Physics in the Life Sciences and Experimental Biology (CoMPLEX), University College London, London NW1 2HE, UK

## Abstract

Autism has been attributed to a deficit in contextual information processing. Attempts to understand autism in terms of such a defect, however, do not include more recent computational work upon context. This work has identified that context information processing depends upon the extraction and use of the information hidden in higher-order (or indirect) associations. Higher-order associations underlie the cognition of context rather than that of situations. This paper starts by examining the differences between higher-order and first-order (or direct) associations. Higher-order associations link entities not directly (as with first-order ones) but indirectly through all the connections they have via other entities. Extracting this information requires the processing of past episodes as a totality. As a result, this extraction depends upon specialised extraction processes separate from cognition. This information is then consolidated. Due to this difference, the extraction/consolidation of higher-order information can be impaired whilst cognition remains intact. Although not directly impaired, cognition will be indirectly impaired by knock on effects such as cognition compensating for absent higher-order information with information extracted from first-order associations. This paper discusses the implications of this for the inflexible, literal/immediate, and inappropriate information processing of autistic individuals.

## 1. Introduction

Autism spectrum disorders are characterized by the development of atypical information processing in diverse areas of cognition. A triad of domains have been identified: limited communication, impaired social interaction, and repetitive behavior/restricted interests [1]. Also the lack of a spontaneous capacity to attribute mental states to others has been proposed to be central [2, 3]. Other problems can exist that disrupt the sensory and motor faculties in individuals with autism [4–9]. 

The etiology that might underlie these diverse and apparently unconnected impairments remains unknown, with the present view being that either a single cause does not exist [10, page 283], [11, page 875] or that the condition is a collection of unrelated cognitive defects [12–15]. Another approach is that autism has heterogeneous etiologies that could underlie an “unified theme” of cognitive impairment [16]. Atypicality suggests that the cognitions of those with autism contain normal or potentially normal components but these, due to some missing aspect in their maturation, develop with an abnormal information processing trajectory. Attempts to understand autism therefore will be closely linked to the understanding that exists upon the components that make up normal cognition and the trajectory of their development.

At present, many aspects of normal cognition have been suggested to be impaired in autism that could potentially affect diverse faculties. These include deficits in executive functions [17–19], inner speech [20], cognitive control over inhibition [21], mirror neurons [22], action monitoring [23], procedural and implicit learning [24, 25], consolidation of experiences [26], prioritizing dynamic stimuli [27], relational processing [28], attentional windows [29], complex information processing [30], formulating and using higher-order rules [31], hierarchical organization in processing information [32], diachronic thinking [33], and temporal cooccurrence, integration, and binding [34, 35]. It has also been suggested that autism is affected by stimulus overselectivity [36], hypersystemising [37], and a greater inference in the local-to-global direction than in the global-to-local one [38].

### 1.1. Autism and Context Impairment

The processing of context is another aspect of cognition that has been proposed to be impaired in autism. Such a context impairment is the basis of the weak “central coherence” approach [39–41]: according to this, normal cognition depends upon a “built-in propensity to form coherence over as wide a range of stimuli as possible, and to generalize over as wide a range of contexts as possible” [42, page 159]. Central coherence, moreover, provides “the everyday tendency to process incoming information in its context—that is, pulling information together for higher-level meaning” [43, page 217]. Those with autism have weak central coherence, and due to this: “Normal children everywhere do well when they understand and take account of context. This is not the case in autism” [42, page 142]. 

The inability to process context in those with autism has also been observed more generally, for example, Hugh Morgan states: “in autism the prerequisites for creativity are not present. The adult with autism cannot extend the known, or bring together understandings to create new ones, because the known remains confined to the specific context in which it was learnt” [44, pages 78-79]. Dodd has also observed: “A child with autism who is told to “pull up your socks” or to “shake a leg” is unlikely to understand that these phrases have multiple meanings depending on context. The child is likely to interpret the phrases literally, regardless of context.” [45, page 159]. An inability to experience things normally in context was also implied in the first description of autism by Kanner [46, page 246]: “A situation, a performance, a sentence is not regarded as complete if it is not made up of exactly the same elements that were present at the time the child was first confronted with it. If the slightest ingredient is altered or removed, the total situation is no longer the same and therefore is not accepted as such…”

Supporting this general link between autism and context, research has found deficits with those with autism in the processing of context for social cues [35], face processing [47], memory [48, 49], perceptual groupings [50], distinguishing essential from variable aspects in event schemas [51], and also in the skills of generalizing from structured settings to more naturalistic ones which may contain unpredictable and context-dependent interpretational elements [52]. This impaired contextual processing in autism has also been proposed to affect the production and comprehension of language in regard to homophone pronunciation [53–55], lexical ambiguity [56], sentence understanding [53, 55], semantic incongruity detection [57–59], prosody production and comprehension [60, 61], metaphor and metonymy appreciation [62], irony [63], discourse continuity [64], and communication pragmatics [65–67]. Repetitive behaviors in autism have been described as originating in contextual impairments, as such behavior is “an assemblage of behaviors defined by their topographical similarity across contexts, inappropriateness, and repetition” [68, page 959]. Individuals with autism, in addition, show superior performance on tasks that require ignoring irrelevant context [69, 70].

### 1.2. Context and Autism Research

A major block upon understanding this link between context impairment and autism is uncertainty about the exact nature of the role which context plays in normal cognition. The word, “context” itself, moreover, is somewhat unclear as to its exact sense and can be understood in various and sometimes conflicting ways. This limits research.

Etymologically the word “context” originates from the Latin words *contex* and *contextus* which refer to when things “weave together”, “interweave”, and “join” [71, 72]. This sense of “weave together” was generalized from physical entities, like textile threads, to include the coherence links between different word meanings in written composition by Roman rhetoricians such as Marcus Fabius Quintilianus [72]. Biblical scholars later treated context as the “co-text” between the parts of a text that occur before and after words [73, 74]. The idea of context has in the twentieth century become further generalized—with context being extended to refer to the “weaving together” of psychological- and psychological-related entities as studied in cognitive science [75], artificial intelligence [76], computer interface engineering [77, 78], pragmatics [79], linguistics [80, 81], social theory [82], neurocognitive [83], and social neuroscience models [84]. In psychology, context has been suggested [74] to conceptually relate to the idea of “field” in cognitive styles [85], and also the background in which perceptual entities are interrelated together in gestalt theory [86]. This idea of central coherence links to the processes by which cognition coheres different parts into larger wholes [39–41]. 

In spite of its widespread use, what context exactly concerns as a component of information processing has not been computationally specified and has remained intuitive and informal among psychologists. As a result of this, at present, no general theory of context has been available to those working upon autism to show how it might be computationally reduced to more basic and developmentally important kinds of cognitive information processing. In consequence, it remains unclear what might be impaired in those with autism that could, following impairments in context processing, produce atypical cognitions. In the absence of such analysis, context has come to be used in autism research to refer imprecisely to nearly any global, holistic, or “higher level” aspect that might be impaired in the processing of a situation, circumstance, or spatial configuration. 

The problems caused by this uncertainty can be seen in the attempts to test the central coherence theory of autism. The role it proposes for context has been analyzed with tasks measuring low-level analytic processes compared to high-level global ones [87, 88]. However, since the central coherence theory describes context in an intuitive way, no objective way exists of knowing if such tests of low-level analytic and high-level global processes do in fact test its suggested disruption to context processing. Another approach [89] has been to use a semantically related visual context priming task [90], but the role of context in this task is judged on intuitive not computational grounds. This suggests there is a need for less informal ideas about the role and nature of context in cognition.

This paper examines the role of context in cognition within an information processing perspective. This is done particularly in regard to how context processing might be impaired in development. Computational modeling in the form of latent semantic analysis research [91, 92], (see also [93]), has recently linked context to the extraction of the information contained in the higher-order associations. This work provides not only a new understanding into the nature of context but raises the possibility (for computational reasons) that context information processing might be particularly vulnerable to selective impairment. Further, the effects of such selective impairment would be the pervasive development of atypical—but not necessarily globally impaired—cognition.

## 2. Cognition, Situation, and Context

### 2.1. The General Problem of Cognition

Cognition can be considered to be the information-processing stage that follows the sensory impressions made by perception. Prior to cognition, sense organs and the initial analysis of their input by sensory cortices separate out information about the world into cooccurring entities [94]. These entities are arranged in their input, by this perceptual separation, in regard to circumstances. As James noted perception has to separate the world in this way into entities as the mind, “which has not yet experienced them separately” or otherwise the contents of the world would be a “great blooming, buzzing confusion” [95]. 

Such sensory circumstances about entities, however, only provide very limited information about the world. Enriching this perception is the task of cognition. Information exists that can be added to the experience of entities provided by perception. This information exists because entities in sensory impressions do not cooccur together at random. This creates hidden information about them that can be extracted from past experience. Individuals have much of this information available to them since they are constantly engaged in perception for about 5,000 waking hours each year. This provides their brain while developing with tens of thousands of different and association rich episodes. Accumulated all together, they contain considerable information with which to create cognition.

### 2.2. Awareness and Apprehension

Cognition enhances the experience of entities in two way, first, by creating an *awareness* of the *situation* of perceived entities and second by creating an *apprehension* of them in their *context*. Essentially, the difference between situation and context is that the awareness of an entity's situation concerns its properties and how they link it in an episode with other entities. The apprehension of an entity's context, in contrast, concerns how the other entities in the episode and their properties cohere together and interweave in regard to that entity. 

To illustrate, we start with perception. Imagine being in a dining room and seeing or feeling an apple in a fruit bowl. This sensory impression tells us that the apple is located within the bowl. This informs us about the circumstances of its location relative to other entities—above, below, adjacent, or far away. This information comes with the processes that create the sensory input. 

Shift to having a cognitive awareness of that apple: it is now experienced in its terms of properties such as its organicness, edibility, heaviness, and inanimateness. This makes it different as an entity to that of a small helium balloon or a resting bird in a bowl, even if these might share similar circumstances in the bowl to the apple, since unlike these the apple will not drift or fly off. On the other hand, we experience the apple, unlike a balloon or bird, as an entity that might soon decay or get eaten. These properties apply to apples generally wherever they are perceived to be located, whether in a room or an orchard, a fruit bowl or on a kitchen table. These properties are, however, experienced as important to its particular situation in the dinning room—its heaviness lets us know that unless moved by some force, the apple will remain in the bowl: its organicness that it may, if left, decay, or be picked up and eaten. 

Now consider the cognitive apprehension of the context of that apple. When so apprehended, the apple is experienced cognitively in terms of its interconnectedness. The apple is given a role by that context such that it could be substituted, and so preserve that context, by putting another fruit, another type of food (such as a candy bar), or even a wax replica of an apple in the bowl. Which properties and therefore which kind of entity depend upon what else is in the dinning room, and why it is there. These might fit in with it having the role to provide food for a guest, or it might be to provide ornament as part of the room's décor. Awareness concerns what is useful to know about an entity that is independent of its circumstances with other entities but which in its situation forms part of its relationship with them—like spokes from a wheel hub. Apprehension concerns the opposite: knowing what is salient about an entity which is dependent upon its circumstances—those ways in which it is contextually interweaved with links that fit it in as part of an episode with the other entities—spokes that radiate from them to it.

### 2.3. First- and Higher-Order Associations

Situation awareness and context apprehension use two different types of information that are both found in past episodes. 

Situational awareness is based upon the information obtained from first-order associations. These exist due to entities being found together—or not—across episodes. Take the example of words. Words occur with different frequencies next to each other. These can be high such as with “soap” and “wash”, or low, such as with “soap” and “aardvark”. Across many such episodes, the presence of one entity may or may not associate with the other. They may be cooccurring such as when one is present, the other is often or nearly always present. Or conversely they may be nonoccurring, where one is present the other is never or only rarely present. When we read the word “autistic”, we are not surprised when it is followed by the word “individual” though we would be if it was followed by the word “goldfish”. It is possible, even without computers, to manually count different words and find how often they appear near to each other. A person needs only to tabulate entities and episodes and extract the statistical patterns of association. These frequency associations have been studied in the past and are the basis of research [96, 97]. 

**Figure 1:**
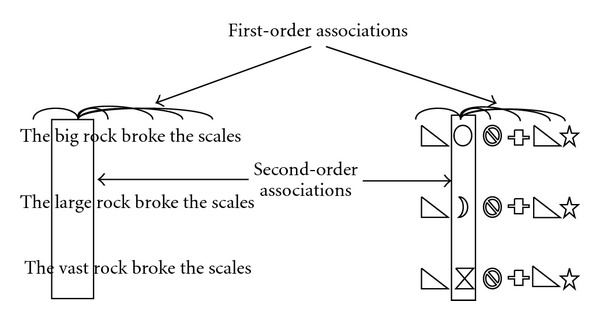
On the right, there are three synonymous sentences that describe the same information chunk. On the left, three different synonymous episodes are made of various shapes. The relationship between synonyms in both groups is illustrated by vertical bands. The word “big”, for example, does not directly associate with “large” but indirectly by appearing in groups with similar words. As illustrated on the left, there is no reason that the higher-order synonymic information which underlies words need be confined to linguistics since first- and higher-order associations can also exist between entities (in this case, shapes) that get grouped into episodes.

Contextual apprehension is based upon the information contained in higher-order associations. These are the associations that exist between entities through indirect associations mediated via other entities (for the difference between the two types of association, see Figure 1). The idea of higher-order associations can be grasped by the example of synonyms. Consider “big” and “vast”; they rarely appear directly together. People normally do not say things like “the big and vast stone broke the wagon”. If one of the words is used, the second is a repetition, and so redundant. Nonetheless, the occurrence of one word contains much information about the occurrence of the other. If “big” appears frequently with another word, say “stone”, so predictably will “vast”; if “big” does not appear with this word, then neither will “vast”. What links “big” and “vast” is not a direct association but having the same kind of direct associations to other words. After all, it is often a matter of indifference as to whether “big” or “vast” gets picked for contextual use in any particular sentence to indicate large size. Such higher-order associations exist not just between “big” and “vast”, but also (with varying strength) between every other possible pair of words, and so occur in regard to everyone of the tens of thousands of words used in spoken or written vocabulary. These shared associations create at a higher level, a strong but indirect link between “big” and “vast”.

Higher and even more indirect levels of cooccurrence word association also exist. Some of the words with which “big” associate, coassociate with it more or less strongly in the presence of yet further words. Thus, if the word “big” appears in a sentence with “stone”, then it is likely that “heavy” will also appear. 

Historically, the existence and importance of higher-order associations have only been recently recognized with the development of computer technology and the use algorithms that can extract the information they contain. As a result, there is a disparity between work upon cognition and first-order associations and that with higher-order ones. A large philosophical literature exists based upon associations and their relationship with cognition, originating in the work of people such as the eighteenth century philosopher David Hume. This deals with cognition as something that arises from experienced direct associations. In contrast, the only work so far upon cognition and higher-order associations is that done by those that have explored its extraction in the field known as latent semantic analysis.

## 3. Latent Semantic Analysis (LSA)

Latent semantic analysis (LSA) can be viewed scientifically as having two parts. First, it offers a mathematical account of the presence and extraction (by the singular value decomposition of a matrix using various transformations into a multidimensional vector space) of the information latent in the higher-order associations contained in past word usage [91, 92], (see also [93]). Second, LSA provides a successfully tested simulation of the use of such information in regard to the many aspects of language such as word sorting and category judgments, estimations of passage coherence, and the quality and quantity of knowledge contained in student psychology essays [91–93, 98]. In particular, LSA shows that children possess sufficient information (contained in the higher-order association of known words) to guess what unknown words might mean given the context information provided by surrounding words [91]. This has allowed LSA to model how children learn the meaning of unknown words, an aspect of child development that prior to LSA was notable in being difficult to explain [91]. 

LSA mathematically uses latent class analysis [99, 100]. So far it remains the only model using this mathematics that has been widely and successfully tested against real data. In the following discussion, because of this, though the principles it uses are more general, the focus is upon LSA. Moreover, it should be appreciated that LSA and the field associated with it of computational semantics are undergoing development. LSA also has its limits, notably that it does not extract all potentially useful information [93, 99, 101, 102]. For example, simplifications in its implementation result in it ignoring much of the information available to word learning such as syntax, word order, and real-world associations [98], nor do the episodes it processes correspond exactly to those of sentences. Implementations that incorporate some of this information are being developed such as those that involve generating “probabilistic topics” [99, 102]. 

Historically, LSA originated in computer scientists seeking an automatic means to retrieve documents by keywords [103–105]. (For this reason, it is sometimes called latent semantic indexing, LSI.) Computer scientists faced the problem that the link between keywords and the words in sought-after-documents depended upon their context. If a searcher types in, for instance, the keywords, “film” and “Marilyn Monroe”, he or she seeks to retrieve not only documents that mention “film”, but also related synonyms found in the same context (such as “movie”, “Hollywood”, and “motion picture”). Further, they want to retrieve only those documents that contain the word “film” that fit in with the context of “Marilyn Monroe”, and not ones containing “film” when it means “thin coating”. Computationally, the context sensitivity needed to identify such synonyms and homograph meanings cannot be reduced to the information contained in its direct cooccurrence associations with adjacent words [96]. Computer scientists to overcome this developed methods to determine the synonyms and homographs of keywords by extracting from large corpuses of texts the higher-order (or indirect) associations that words have with each other. This information had not been previously investigated, as previous work upon the information in texts (due to limits upon computer power) was confined only to the extraction of first-order or direct cooccurrence associations. Using information extracted from higher-order associations, LSA applied to word searches has been able to efficiently detect synonyms and homographs needed for keyword document retrieval programs [103]. Although commercial confidentiality prevents the open publication of the implementation of contemporary search engines such as Google, web newsletters (such as http://www.free-seo-news.com/newsletter147.htm) report they incorporate, at least in part, the use of LSA-derived context extracting techniques.

### 3.1. LSA, Context, and Meaning

LSA provides an empirically tested account of the phenomena of context and what might be called “meaningfulness”. There are two issues here: meaningfulness as a measurable “behavioral” aspect of words and the ability of LSA to match the performance of human individuals on this measure. 

Context and meaning link through “behavioral properties”. These concern not intuitions about meaning but behavior as shown in human judgments about word meaning. These properties contrast with the “sense” properties traditionally investigated by philosophers that concern intuitions about semantic reference [106]. Behavioral properties concerning the comprehension of meaningfulness affect the objective behavior of human judgments about words in terms of their similarity, their intersubstitutability in sentence contexts, and their predictability in certain sentence contexts [80, 81, 107, 108].

#### 3.1.1. The Similar-Dissimilarity Property

All semantic words have various degrees of similarity and distance of meaning. “Vast” and “big” are regarded as having a great deal of similarity and closeness, “vast” and “small”, a smaller amount, while “vast” and “kiss” are very distant, for example. What might underlie this property of semantic similarity is uncertain: the resemblance or lack of meaning between words cannot be inferred from their visual or sound identity nor their close association with other words (the level of first-order associations). For example, nothing about the perceptual nature of “vast” and “big”—its letters and phonemes—provides information that they are synonyms. Nor is this information provided by their immediate associations with surrounding words in the sentences containing them [96]. In spite of this, humans have an immediate apprehension of which word meanings are similar and which are not.

#### 3.1.2. The Synonymy Intersubstitutability Context Property

*“*Meaning” has the property that words that have roughly the same “meaning”—synonyms—are contextually intersubstitutable [109]. The more similar the meaning, the more alike the contexts in which they appear [80, 81, 107, 108]. Consider “vast” and its synonyms, “big”, “large”, or “huge”, each of these three words can be substituted in most sentences without significant change of sentence meaning. “The big rock broke the wagon”, for example, means roughly the same as “The large rock broke the wagon”, and even, “The vast rock broke the wagon” (see Figure 1). Like the distance-similarity property, while the human brain can understand intuitively which words can be swapped, philosophical or other analysis finds it difficult to specify the nature of the processes involved.

#### 3.1.3. The Predictability Usage Property

Linked to context is the property that words do not appear at random in normally encountered sentences. Expectations and constraints exist in the form of word usage patterns, about which individual words tend to appear with which other ones [110]. This can be experimentally shown with cloze sentences in which single words are omitted and individuals face multichoice options to pick the missing word [111]. If human cognition is presented with the incomplete sentence, “The…stone broke the wagon”, and asked to predict the most likely missing word out of “big”, “forgetful”, and “sweet”, the word picked would be “big”. “The big stone broke the wagon” has an expected pattern of usage, that is, absent—even though they are readily comprehendible—in the sentences, “the forgetful stone broke the wagon”, and “the sweet stone broke the wagon” (understandable, e.g., if they were to appear in a “fairy story”). Indeed, even without suggested word choices, if single words are cut from writing, human subjects can guess with the remaining before and after words (depending upon sentence position) up to half of them ([112], Figure 1). Further, if the next word in a piece of ordinary writing is covered, and an individual has not read ahead, they will be able to guess it in about one in four times [110, pages 91-92]. The ability to fill in missing words in sentences is used in the testing of the comprehension progress of language learners [113], and the assessment of language competence, for example, in TOEFL (Test of English as a Foreign Language) certification.

### 3.2. Semantic Space and LSA

Judgments about semantic closeness cannot be made directly upon the information extracted from higher-order associations. The information this contains about words therefore needs to be changed into a form that can be compared with human judgment behavior. This is done in LSA by converting the information that associates words into a multidimensional vector space. In this space, synonyms occupy the same locality (due to their intersubstitutability in any context), while the different meanings possessed by heteronyms (homographs and homophones) are widely separated (they produce different meanings when put in different contexts). More generally, the closer the meaning of words (in terms of cosine similarity), in this space, the closer mathematically are their vectors, and *vice versa*. This provides a means to assess semantic closeness created by LSA since such measures can be matched against judgments made by human subjects.

In this multidimensional space, vector locations not only are given to words but also to complete and incomplete sentences. A word can be synonymous, after all, not only with another word, but a group of words—for example, the definition “being of extreme size” is synonymous with “vast”. Sentences and other groups of words are given a vector location by mathematically adding together all the vectors of their individual words [91–93]. This provides a new vector location that is a kind of mathematical “center” of their individual vectors. This turns out to be useful as a means of gaining information about unknown words. This is because the words that contextually surround the unknown word due to the coherence contained in their higher-order association information provides information in regard to the unknown word.

### 3.3. LSA and the Behavioral Properties of Meaning

LSA successfully models the behavioral properties of meaning, such as similarity and the distance judgments, and does so with a performance that matches that of human subjects [91, 92]. It also models successfully the ability to fill in words in TOEFL. For example, applicants to US colleges with English as a second language if given a word and four possible synonyms will get 64.5% correct; the LSA model, 64.% [91, page 220]. 

As a consequence, LSA provides strong support to suggest that the higher-order associations which it extracts play a key role in the brain's generation of the context that underlies the meaning of words (as reflected in the brain's making of similarity and dissimilarity judgments). This would be an unlikely finding if the higher-order associations extracted by LSA were a mere epiphenomenon to the brain's processing of meaning. 

Landauer and Dumais [91] further find that the ability to infer unknown words from the context of known ones, in the high-dimensional space generated from higher-order associations, plays a key role in language development. A key theoretical problem in child development is explaining the success of children in learning new words. Children learn on average 10 to 15 new word meanings each day but only one of these words can be accounted for by direct instruction [91]. The other nine to 14 word meanings need to be picked up from another source of information. Landauer and Dumais [91] have found that when children encounter an unfamiliar word, the context of its surrounding known words contains sufficient information to enable them to guess its likely meaning. The words children already know together with the information associated with them from their higher-order associations allow them to create a semantic space from the words near the unknown one. These words each have a vector location in this space that can then be added together to create a vector of the context of the unknown word. This vector turns out to be sufficiently near to the meaning of the unknown word to provide a source of information for determining what that might be. The ability to learn new words expands with vocabulary and language experience. LSA explains why: the more words an individual knows, the better the multidimensional semantic space they create, and so the easier it is to use the information surrounding words to learn new ones [91].

The LSA computer model shows that most of the information needed to make judgments about meaning and learn new words comes from higher-order associations not first-order ones [91, page 226]. Unlike first-order ones, higher-order associations tend to provide only relatively weak information about word usage. However, higher-order associations are by many orders of magnitude much more numerous than first-order ones, and so (when added up) in total contain much more information. Indeed, as Landauer and Dumais note, “About three quarters of LSA's word knowledge (when tested) is the result of indirect induction, the effect of exposure to text not containing words in the tests” [91, page 226].

The nature of the information identified by LSA as central to language and word learning is contextual. This raises the question of whether the traits identified with the extraction of higher-order information by LSA that underlies this might aid the understanding the context deficits found in those with autism.

## 4. Word Meaning Atypicality in Autism

### 4.1. Language Context Impairment and Autism

From Leo Kanner [46, 114] to the *Diagnostic and Statistical Manual of Mental Disorders. Fourth edition*, [115], language and meaning problems have been central to the diagnosis of autism. They include mutism, echolalia, language acquisition delay, pedanticism, and atypicality in the understanding and use of word meaning. The latter will be of concern here. Kanner [114, page 243] noted that, “the autistic child has his own private, original, individualized references, the semantics of which are transferable only to the extent to which any listener can, through his own efforts, trace the source,” and that their word meanings are “rooted in concrete, specific, personal experiences of the child who used them” [114, page 243]. Similar comments have been made by Frith and Happé [41]. They note that children with autism “may use single words in a simple, associative way, so that “Apple” always means, “Give me apple”. The single words acquired are often esoteric (e.g., “Beethoven”) and not like the first words of a normally developing pre-schooler. Neologisms (e.g., “bawcet” for bossy), or familiar words with special meanings “yes” meaning “carry me on your shoulders”), also reflect the very concrete context of word and object”. These comments suggest that individuals with autism when they hear unfamiliar words are restricted in their attempts to understand what they might mean to the use of their immediate (and often misleading) associations in the physical world. This, indeed, was described by Kanner [114, page 242] in regard to a child called Paul G who said “Peter eater” whenever he saw anything resembling a saucepan. According to his mother, when Paul was two years old, while busy in the kitchen, she was reciting to him the nursery rhyme about “Peter, Peter, pumpkin eater”, when she dropped a saucepan. Ever since Paul has understood “Peter eater” atypically to mean “saucepan”. He had made an association between “Peter eater” and the most salient event at the time of its been said—the dropped saucepan. 

This bias in those with autism to using direct cooccurrence associations would suggest a particular kind of failure in word learning: first, that they fail to employ the context-based cognition processes by which nonautistic individuals acquire word meaning; second, that in its absence, they rely upon the accidental nature of word and event cooccurrences to guess what words might mean; third, that when they have done this, they are impaired in using context to appreciate the inappropriateness with which they have understood a word. This has two results.

1. It delays learning words, since it removes the main source of information by which individuals usually acquire a word's meaning. This could explain why some individuals with autism are mute or echo words and phrases—a word usage that does not require that they understand a word's meaning.
2. That when they do acquire language, this could explain why they tend to pick up bizarre and restricted ideas about the meaning of words: without an ability to use context to judge what an unknown word might mean, they are limited to its direct associations with events and situations. This could also be a factor in the pedanticness with which words are understood [116], and the difficulties in understanding words as metaphors [117] and idioms [118].

### 4.2. Autism, LSA, and Context Impairment

#### 4.2.1. Homographs

The miscomprehension of homographs by those with autism provides evidence of a deficiency in the processing of context as modeled by LSA. Four sets of experiments [53, 54], [55, experiment 1], [89, experiment 4] but see experiment 2 in [119] involving those with autism that can read have found evidence that they ignore sentence context when asked to pronounce a homograph. Unlike those without autism, they tend to pronounce the most common occurrence of homographs, rather than the one that fits its sentence context. Thus, they will tend to pronounce the word “tear” in the sentence, “The lady had a TEAR on her dress” in the sense of a cry droplet rather than of a rip or cut. This suggests that they might not access the context used by nonautistic people in word comprehension. LSA has shown that the context needed to disambiguate the different meanings of homographs is extracted from higher-order associations.

#### 4.2.2. Coherence

LSA has also shown that context information is used by people to judge the degree of coherence present in texts [120, 121]. Thus if LSA-like processes were damaged in autism, the perception and use of such coherence would be expected to be atypical. Evidence suggests that this is the case: individuals with autism tend to fail to comprehend the normal coherence that allows inferences between sentences [55, 122]. In further support of this, DSM-IV and others [46, 64] note that those with autism seem unable to create continuity between sentences, they fail to continue the topic of conversations, and instead, regardless of meaning, repeat words or phrases.

### 4.3. Atypical Cognition and Autism

The language impairments of those with autism are consistent with an impaired ability to extract and use the higher-order associations modeled by LSA. But autism also affects other aspects of cognition in addition to language. Could a similar defect exist in the use of the information contained in higher-order association between the entities they process? Landauer and Dumais note that the information processing principles behind LSA are not domain specific.

There is no reason why much more complex structures, with mental (or neural) events at varying temporal scales and various degrees of repeatability could not exploit the dimensionally matching mechanism to produce similarities and generation among and between psychological entities of many kinds [91, page 228].

There is thus no reason why these “psychological entities of many kinds” should not also exist in the diverse variety of nonlinguistic cognitive faculties. It is not implausible that other cognitions might not use similar processes to those described by LSA for language. 

First, three key things underlying LSA—the information contained in higher-order association, context, and intersubsitutability—are not specific to language. Like words, entities identified in nonlinguistic domains can also have cognitive attributes akin to “synonymy” and “heteronymy”. A car, a donkey, and a sedan-chair may look very different, but they provide similar intersubstitutable means of travel when on a holiday to get from one place to another. They—at least if our desire is holiday transport—can substitute for each other (much as the words, “vast”, “big” or “being of extensive size” can in the same sentence substitute for each other). Here, however, what they share is not the same meaning but the same functionality as required by our needs and our cognition of those needs. “The tourist went in a car to the shops,” “The tourist went on a donkey to the shops,” or “The tourist went in a sedan-chair to the shops” while different as physical activities are similar in terms of their being solutions to a holiday need to travel. (And, reflecting this, the sentences describing them mean roughly the same in that context.) Likewise, in terms of function, an entity can be “heteronymous” in regard to different cognitive relevancies: a car in one context can be a means of transport, in another a place to escape rain, and in yet a further one, a status symbol. It would thus seem plausible to infer that the multidimensional space LSA found for words and meaning has its parallels for entities in other domains.

Second, the problem of identification of unknown entities from context is not confined to words. A cognitive problem faced by many faculties is identifying something—a hidden object, an unfamiliar response, or an unidentified aspect of a situation—given the information provided by surrounding known events and entities. This problem is encountered particularly by the faculties that provide humans with a sense of security, social interaction, and the apprehension of mental states. Such faculties develop using the information contained in the tens of thousands of episodes encountered in daily experience across the many years in which cognition matures. Thus, such faculties have available the higher-order association information contained across such episodes to generate a multidimensional space that can be used to identify unknowns from their surrounding context. 

Context information processing also plays a central and crucial role in behavioral adaptability. This depends upon several skills.

1. Episodic inference: the apprehension of the properties of entities that might be missing, hidden or unfamiliar in an episode. Without episodic inference, it is difficult to learn about the properties of new useful entities.
2. Episodic equivalence: apprehending that episodes are equivalent (or near substitutes to past ones) in their properties even though they consist of different sets of entities. Without episodic equivalence, it is difficult to grasp that unfamiliar situations are the same as familiar ones in spite of superficial first-order differences.
3. Episodic reduction: apprehending which entities in episodes are redundant and so can be removed. Without episodic reduction, it is difficult to learn more efficient or economical ways of being aware, attending, communicating, achieving goals, or performing tasks. For example, efficient communication depends upon skill in being elliptical about information that the other party already knows or can readily infer.

This raises the possibility that the extraction of context information from higher-order associations and its following consolidation into cognition might be widely exploited by faculties other than language in their own development and function. From this, it follows that impairments to such processes might produce impairments in these nonlinguistic functions.

## 5. General Atypicality Traits in Autism

The etiology of autism has four characteristics: (i) it affects diverse cognitions, (ii) it produces cognitions that are atypical, (iii) it allows for isolated nonretarded or even supercompetent abilities, and (iv) it is heterogeneous. A developmental condition with these characteristics could be expected to arise following impairments linked to the extraction of information from higher-order associations. This is because such processes could be expected to be carried out by specialized operations that are separate to those underlying the cognitions that utilize this information. Another related possibility is that impairments might happen to the consolidation of such information into cognition. This separation of the processes that extract and provide information from the cognitive processes that use it would allow for the latter to be intact—except for its incorporation of this higher-order information. This would result in atypical but not necessarily retarded cognition. This might occur due to the lack of such information causing changes to cognitive processes such as when they compensate for missing higher-order information with that extracted from first-order associations. This separation between extraction and the utilization of information would also favor the specialization of such information extraction and its shared use by several distinct domains of cognition. This separation, moreover, would allow the existence of impairments of different types arising at the stages of extraction, transmission, and consolidation. 

### 5.1. Diversity and Separated Shared/Modularized Neural Resources

The information contained in higher-order associations is more difficult to extract than that in first-order associations. To extract the information contained in the first-order association between two entities requires relatively easy computation since it involves just those episodes that contain them. Moreover, it is processed as a normal part of cognitive activity. As a result, it can be acquired directly by the cognitions utilizing such information from the experience of such entities in past episodes. But to find the information contained in higher-order associations requires processing at a global level of all entities in all episodes as a totality, something that is not normally done as part of cognition. 

Another difference is that the extraction of information from higher-order associations concerns very weak bits of information that accumulate into useful information due to the very large numbers of associations containing these slight bits of information in past episodes. Further this weak information is scattered across the brain. Different parts of the brain, for example, process the “noun” and “verb” qualities of entities [123], and also the concepts of tools and animals [124]. In consequence, the information in the higher-order associations will be spread and hidden across the brain. This will need a process that can extract it irrespective of where it is neurologically located. These computational factors of global, weak, and neurally dispersed information require that the extraction of the information from higher-order associations will be specialized since only this will allow it to be effectively and efficiently processed by the brain 

There is also the factor that many cognitive faculties develop in regard to overlapping sets of experiences. For example, the ability to contextually apprehend security and danger derives from past experience in terms of their safety and danger. These episodes are also ones that contain context information needed for apprehending context in language and social interactions. Thus for distinct functions, the same or overlapping episodes will need to be processed to extract context information from higher-order associations. This creates a computational redundancy that can be avoided if one process extracts this information, and then it is transferred for separate consolidation into different cognitions. 

 Such separation and specialization is consistent with how the brain is internally organized. Information processing in the brain occurs in white matter circuits through which information is transferred, modified, and consolidated. This consolidation/transfer process occurs for the cerebral cortex in regard to modification of its information by white matter traits to and from the cerebellum [125], hippocampus [126], and basal ganglia [127]. Splitting information processing in this way allows areas to specialize in different types of computations.

Consistent with this, a consolidation processing defect has already been suggested to underlie autism in regard to the hippocampus and cerebral cortex and cognitive representations of the environment acquired from experiences [26]. In addition, impairments in white matter integrity have been identified in those with autism that impair the connectivity needed for such modification and consolidation [128, 129].

### 5.2. Atypical but Not Globally Impaired Cognition

 The separation in the brain between the processes that extract higher-order information and the cognitions that use this information has important implications for the etiology of its possible impairment. This is because the impairment of such separated and specialized processes could occur without impairment occurring directly to the processes that use extracted contextual information. Their impairments, however, would still have a knock-on impact upon the processes underlying normal cognition. This is because these otherwise intact processes would be disrupted by the consequences of them not developing with the information extracted from higher-order associations.

This would greatly alter these processes. First, many cognitions would not be able to carry out the functions that required the information extracted from higher-order associations. Second, while many normal cognitions would be impaired by the lack of information from higher-order association, some which use only first-order information would not be affected creating an uneven cognitive development. Third, some cognitions might compensate for missing information with that extracted from first-order associations functioning creating forms of cognition not found in unaffected individuals. Fourth, in rare cases, the lack of information from higher-order associations will result in the processes acquiring greater competence compared to that present in neurotypical individuals. 

 Thus, following an impairment to the processes extracting the information from higher-order associations, many faculties would be unimpaired (those not using this information), some impaired (those using it and for which the information from first-order associations cannot replace), some atypical (those compensating the missing information from higher-order associations with that from first-order ones), and some superior (those cognitions impaired by the information from higher-order associations). This would parallel the symptom profile of autism where many cognition competences are preserved though many are impaired—for example, having reading comprehension impaired but not the ability to read, spell, and do computational tasks [130] and mechanical reasoning that is preserved compared to social reasoning [2, 3].

### 5.3. Heterogeneous Development

Autism takes many forms and any model must be consistent with such diversity. The extraction of information from higher-order associations is part of a circuit with several stages. The circuit contains the acquisition of experience in terms of episodes containing associations, its transfer through white matter tracts to specialized processes that extract the information in regard to its higher-order associations, the transfer of this information by white matter tracts to cognition, and finally its consolidation as context into cognition. Each of these might be separately compromised with different consequences. For example, impairment to the extraction process would result in widespread impairment, while impairment to white matter tracts might limit impairment to only those cognitions for which they provide a specific information transfer. 

Differences could also occur within the specialized computational process. For example, varying the size of the window of association elements from which such information is extracted, and the number dimensions into which such extracted information is rerepresented might produce different kinds of atypicalness. These offer diverse ways in which impairment might create different forms of contextual information deficiencies.

Another layer of variables on top of this would be created by the different environmental and educational support that ameliorates or increases their impact on such neurocognitive impairments. The neurobiological factors that cause impairment upon neurocognitive processes, in addition, could independently of this be expected also to have their own separate effects upon cognitive integrity (such as causing general mental retardation and epilepsy). Thus, no reason exists to assume (except in broad aspects) that in different individuals the behavioral consequences of an impairment to processing higher-order associations, and so context, will in detail be alike.

## 6. Discussion

The LSA model offers a tested computer model of the role of context in comprehending the meaning of words. It identifies that cognition depends not just upon the information extracted from direct (first-order) associations but also, and critically, that extracted from indirect (higher-order) ones. First-order (direct) information and higher-order (indirect) information are, however, very different in that they make different contributions to cognitions. Notably, the apprehension of an entity's context requires the information extracted from higher-order associations. These types of information, moreover, originate from different processes of extraction. In cognitive processing, the awareness of an entity's situation only arises from the use of information extracted from first-order associations. This raises the possibility that cognition might be vulnerable to selective impairment that does not produce a general loss of cognitive competence but one that is characterized by atypical cognition. This is because the extraction of information from higher-order associations requires specialized and separated processes that consolidate this information into the cognitive processes that utilize it. If these processes are impaired, an individual will retain cognitive competent processes, but only in regard to the information extracted from first-order associations. A feature of autism is an atypically in cognition that is broadly characterized by a lack of context and an overdevelopment in experience of direct associations. This parallels the impairments that might be predicted to follow impairments in the extraction and consolidation of the information in higher-order associations.

There is an important limit. LSA is a recent approach and has not been expanded from language into a general model of the role of context in cognition and development. This reflects LSA being a theory based upon the extraction of information from a large body of episodes. This can be readily done for language since corpuses of texts containing many tens of millions of words exist. Even so its processing of them is preliminary in ignoring many sources of information. Critically, nothing equivalent exists compared to such corpuses of word usage for any other domain of cognition. A child may go through tens of thousands of episodes of interaction with other people, but no objective record of them exists for computational analysis. The work of Landauer and Dumais, as a result, has not been followed by related analysis in other areas such as how human brain's think, feel, and socialize, and how such processes on the basis of the information in higher-order associations are acquired. Context is as important here as it is with language, but the opportunity to computationally analyze it has yet to be developed. 

LSA suggests several areas for future research. One is computational lesioning. This has already occurred for other developmental conditions, such as dyslexia, Williams syndrome, brain injury, and specific language impairment [131–134]. If the general approach of this paper is correct, then lesioning LSA computer simulations should produce atypical word comprehension and learning. Further study could be made in those with autism and other forms of atypical cognition based upon such lesioned LSA models. Models of autism and context have been developed [135, 136]—LSA suggests ways in which this research could be expanded with more specific notions of context and its impairment.

Atypicalness in autism also suggest areas in which the LSA could model uninvestigated aspects of language. For example, the multidimensional space created by LSA represents, in a manner, conceptual knowledge—an aspect that has resulted in LSA models being successfully used to automatically mark the content of student essays in regard to their knowledge of psychology textbooks [92]. The acquisition of theory-of-mind skills is known to link to language [137] and the social exposure to mental state words [138]. This suggests it is likely to be extracted by LSA from the usage of mentalistic words. Theory-of-mind skills have not as yet been modeled by computer simulation, nor has the LSA model been tested as to whether it can simulate them. According to the approach proposed here, the lack of theory-of-mind skills in those with autism will derive, like their other impairments, at least in part, from a defect in extracting and using higher-order associations contained in mentalistic words. Given the dependence of theory-of-mind on language, and its proposed dependence upon higher-order context, this should be modelable by LSA. 

Another area important to future research is developing tasks that can better identify the processes that depend upon the information extracted from higher-order associations. Charles' Sorting Task [107, 108] is relevant here as it directly measures the capacity to comprehend synonyms. In this task, cards are prepared with sentences omitting words which may or may not be synonyms. An individual is asked to go through the cards guessing the missing word and sorting them out into several “word” piles, one for each type of missing word [107, 108]. The discriminability with which words omitted and are sorted into different piles is a measure of their semantic closeness, and an individual's capacity to extract and utilize such context information. This task has so far not been used by autism researchers. The approach given here would suggest such performance in those with autism would be considerably impaired both in tasks using words, and versions in which in the place of sentences, episodes are made up of nonlinguistic entities related to social interaction. 

In conclusion, LSA shows that cognitive science until recently missed the key role of context-based cognition in the domain of linguistic meaning. LSA further shows that this competence derives from the information extracted from the higher-order associations between entities in past episodes. This omission was due to its investigation requiring technology that has only become recently available. LSA shows that in the domain of language, the performance of many tasks can be best accounted for by simulations which employ such information. While LSA is concerned with language, the mathematics upon which is based is not specific to language and could underlie other cognitive domains in which context is processed. There is, of course, a theoretical jump as to whether cognition uses similar processes in nonlinguistic domains. However, in many of these domains both the circumstances are present for such information, and the cognition processing in them shows characteristics—such as the use of context—that strongly suggests that they do indeed employ (or have a need to employ) such information. The problems in extracting information from higher-order associations would suggest that this requires processes separate to the cognitions that use such information. Moreover, that such processes might contribute to different domains of cognition. This raises the possibility of cognitive impairments that are limited to context having an affect upon diverse faculties. Such a pervasive impact would not create a general cognitive impairment but rather atypical cognitions. As shown above such impairment in many respects parallels the traits found in autism. This paper has reviewed this possibility in regard to furthering the understanding of autism.
